# Cleft Palate

**Published:** 1871-05

**Authors:** William J. Fogle


### ARTICLE II.

### *Cleft Palate.*

By William J. Fogle, D. D. S.

Among the many cases I have met with in my practice, perhaps I can find none more interesting than one of cleft palate, which I treated successfully some years ago. It was not one of those extreme, aggravated cases in which an inter-maxillary bone had been formed and left suspended from the vomer, by the failure of the long formation from the anterior part to unite with the main body of the superior maxillary, and accompanied by hare lip; nor was the velum pendulum involved; but there was simply a fissure beginning a line or two behind the incisor teeth and extending along the median line to a point opposite the second molar teeth; this fissure was fully one-third of an inch wide or probably a little wider at the largest part. The teeth in this case were, with few exceptions, good, and had every indication of belonging to those of the first class, though through neglect, imprudence and inattention on the part of the patient, some few of them were much decayed. The patient—a lady of about 30 years of age—objected to the surgical operation of staphyloraphy, and as some of the teeth were so much decayed as to render extraction necessary, I deemed the first operation useless, inasmuch as the plate could be made to serve the double purpose of sustaining the artificial teeth, and also closing the defect in the palate. The patient stated that she experienced much inconvenience from the malformation of the mouth, both during mastication and also in conversation.

After I had removed the decayed teeth and brought the mucous membrane to a normal condition, I took an impression of the mouth in wax; this material copied the various parts and markings with so much accuracy that I was enabled to study the many parts of interest presented, at my leisure. After careful examination I determined to obliterate all traces of the fissure from the model, and to place the teeth upon a gold plate, the retention of which shoud be aided by two small air chambers ; these I made in the form of a crescent and placed one on either side of the fissure so that the points should be opposite each other. The teeth, two central incisors. a first bicuspid on the right, and a sec ond bicuspid on the left, were arranged in the usual manner and the plate inserted. It was very gratifying to find that the plate gave entire satisfaction, the patient having, as it were, been brought in a momont from affliction to perfect health and comfort.

In regard to taking the impression with wax, I must say that if I had possessed at the time a sufficient knowledge of plaster as an impression material, I would have preferred its use, for the reason that I would have found less difficulty with it than in the case of the wax, although with this latter material I succeeded very well.

I could also have dispensed with the air chambers, by having a roughened surface on the plate instead, which is easily accomplished by passing a fine gauze wire on the plate through the rollers. The gauze used for this purpose may be of brass, steel or iron. This plan I adopt altogether whether I use the clasp or not, and I find that it answers an admirable purpose. In many cases of partial pieces where clasps would otherwise be required, experience teaches me that by this process the plate adheres so firmly to the mouth, that there is not the slightest necessity for the use of the clasp. On this point all will agree with me who try this plan and do their work in a proper manner ; and unless the work is done well and a good adaptation secured, it had better not be done at all. While referring to clasps, I will take occasion to say, with all due respect to those who use them, that I do not think that clasps should be employed in any case where their use can possibly be avoided. It matters not how well they may be adapted to the teeth, or how fine the material of which they are composed, they have a tendency to injure the teeth to a greater or less degree in every case where they are employed.

